# Development of pH-sensitive Dextran Derivatives with Strong Adjuvant Function and Their Application to Antigen Delivery

**DOI:** 10.3390/membranes7030041

**Published:** 2017-08-04

**Authors:** Eiji Yuba, Shinya Uesugi, Maiko Miyazaki, Yuna Kado, Atsushi Harada, Kenji Kono

**Affiliations:** Department of Applied Chemistry, Graduate School of Engineering, Osaka Prefecture University, 1-1 Gakuen-cho, Naka-ku, Sakai, Osaka 599-8531, Japan; kk.su.baseball.fan@gmail.com (S.U.); su108056@edu.osakafu-u.ac.jp (M.M.); swb02047@edu.osakafu-u.ac.jp (Y.K.); harada@chem.osakafu-u.ac.jp (A.H.)

**Keywords:** pH-sensitive liposome, dextran, cytokine, cancer immunotherapy, adjuvant

## Abstract

To achieve efficient cancer immunotherapy, the induction of cytotoxic T lymphocyte-based cellular immunity is necessary. In order to induce cellular immunity, antigen carriers that can deliver antigen into cytosol of antigen presenting cells and can activate these cells are required. We previously developed 3-methyl glutarylated dextran (MGlu-Dex) for cytoplasmic delivery of antigen via membrane disruption ability at weakly acidic pH in endosome/lysosomes. MGlu-Dex-modified liposomes delivered model antigens into cytosol of dendritic cells and induced antigen-specific cellular immunity. However, their antitumor effects were not enough to complete the regression of the tumor. In this study, antigen delivery performance of dextran derivatives was improved by the introduction of more hydrophobic spacer groups next to carboxyl groups. 2-Carboxycyclohexane-1-carboxylated dextran (CHex-Dex) was newly synthesized as pH-responsive dextran derivative. CHex-Dex formed stronger hydrophobic domains at extremely weak acidic pH and destabilized lipid membrane more efficiently than MGlu-Dex. CHex-Dex-modified liposomes were taken up by dendritic cells 10 times higher than MGlu-Dex-modified liposomes and delivered model antigen into cytosol. Furthermore, CHex-Dex achieved 600 times higher IL-12 production from dendritic cells than MGlu-Dex. Therefore, CHex-Dex is promising as multifunctional polysaccharide having both cytoplasmic antigen delivery function and strong activation property of dendritic cells for induction of cellular immunity.

## 1. Introduction

Recent success of immune checkpoint inhibitors such as CTLA-4 antibody or PD-1 antibody has increased attention to cancer immunotherapy [1]. The canceling of immunosuppression by tumor microenvironments leads to the activation of cancer-specific immune responses, especially cytotoxic T lymphocytes (CTLs), which play a crucial role in eliminating tumor cells, resulting in tumor regression. On the other hand, it has been also reported that immune checkpoint inhibitors hardly show therapeutic effects on the patients that have no cancer-specific CTLs before treatment of immune checkpoint inhibitors [2]. These facts clearly show the importance of cancer-specific CTL induction along with the treatment with immune checkpoint inhibitors. For induction of cancer-specific immune responses in vivo, the delivery of cancer antigen into antigen presenting cells (APCs) such as dendritic cells or macrophages, which start and activate antigen-specific immune responses are crucial [3,4]. Particularly, cross-presentation, which is an antigen presentation of exogenous antigen via MHC class I molecules, is required to induce CTL and cellular immunity [5]. For induction of cross-presentation, the control of antigen fate in APCs, specific receptor-mediated endocytosis or activation of APCs are regarded as important factors [5]. The control of the intracellular fate of the antigen can be controlled by the design of antigen delivery carriers. Hence, various antigen delivery carriers for induction of cross-presentation have been reported such as polymeric nanoparticles, polymeric micelles, polysaccharide-based nanoparticles and liposomes [6,7,8,9,10,11,12]. Among them, membrane-based antigen delivery systems have advantages to achieve the control of intracellular antigen fate by using biological process such as membrane fusion.

We previously developed antigen carriers to achieve the control of antigen fate within dendritic cells and to induce cross-presentation using liposomes modified with pH-responsive fusogenic polymers [13,14,15,16]. For this purpose, carboxyl group-introduced poly(glycidol)s or polysaccharides have been designed as pH-sensitive polymers [13,14,15,16]. Especially, liposomes modified with 3-methyl glutarylated dextrans (MGlu-Dex) delivered model antigenic proteins ovalbumin (OVA) into cytosol of dendritic cells via membrane fusion with endosomal membrane responding to weakly acidic pH in endosomes [15]. These liposomes induced MHC class I-mediated antigen presentation (cross-presentation), leading to tumor regression in E.G7-OVA tumor-bearing mice [15,16]. However, antitumor immunity induced by MGlu-Dex-modified liposomes was insufficient for complete regression of tumor.

**Figure 1:**
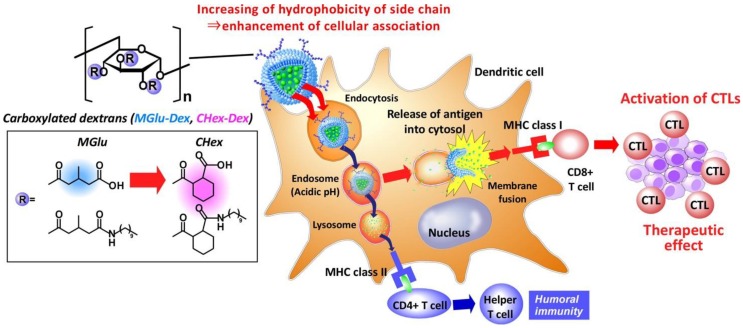
Carboxylated dextran derivative-modified liposomes for intracellular delivery of antigen to dendritic cells. CHex-Dex, which has more hydrophobic side chain than conventional MGlu-Dex, is expected to enhance cellular association and pH-responsive disruption of endosomal membrane of liposomes, leading to induction of cellular immunity.

In this study, the improvement of antigen delivery performance of dextran derivative-modified liposomes was attempted by increasing hydrophobicity of dextran derivatives. CHex unit, which have cyclohexyl group as a spacer next to carboxyl group, was introduced to dextran instead of MGlu unit. Resultant 2-carboxycyclohexane-1-carboxylated dextran (CHex-Dex, Figure 1) was modified onto liposomes. Hydrophobic CHex-Dex is expected to increase cellular association of liposomes and pH-responsive membrane fusion ability compared with MGlu-Dex. Here, the effect of side chain structure of dextran derivatives on their pH-responsive interaction with lipid membrane, uptake by dendritic cells and intracellular delivery performance was examined. In addition, we unexpectedly revealed the quite strong adjuvant function, which is the ability to activate APCs or induce the maturation of APCs, of CHex-Dex compared with MGlu-Dex.

## 2. Results and Discussion

### 2.1. Characterization of Dextran Derivatives

**Figure 2:**
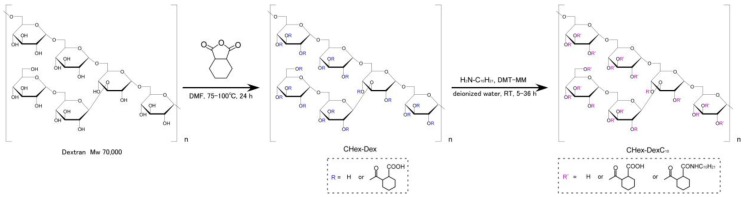
Synthesis of CHex-Dex and CHex-Dex-C_10_.

Carboxylated dextran derivatives (CHex-Dex) with different contents of CHex groups were synthesized by reacting hydroxyl groups of dextran with various amounts of 1,2-cyclohexanedicarboxylic anhydride (Figure 2). Figures S1–S5 depict ^1^H NMR spectra of CHex-Dex. Introduction of CHex groups to dextran was confirmed from the existence of peaks corresponding to CHex groups (1.2–2.0 ppm, 2.6 ppm). From the integration ratio of peaks of CHex residues to those of dextran backbone (3.4–4.2 ppm, 5.0 ppm), the amounts of CHex groups combined with hydroxyl groups of dextran were estimated as shown in Table 1. Decyl chains were further introduced to CHex-Dex by reaction of decylamine with carboxyl groups of CHex units to fix polymers onto liposome surface (Figure 2). Resultant polymers were designated as CHex-Dex-C_10_ and ^1^H NMR spectra for these polymers were presented in Figures S6–S9. From the integration ratio between dextran backbone, CHex residues, and decyl groups (0.9–1.5 ppm), decyl residues and CHex residues combined with hydroxyl groups of dextran were determined as shown in Table 2.

**Figure 3:**
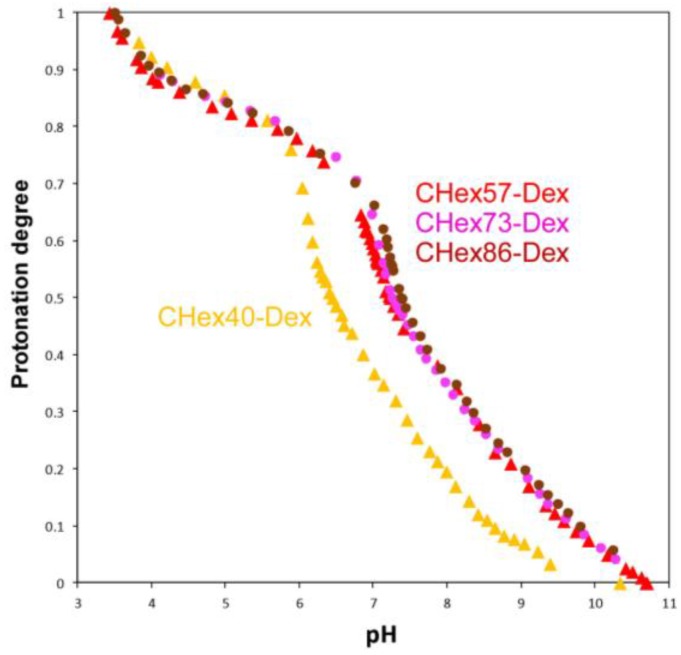
Titration curves of dextran derivatives measured by acid-base titration using a conductivity meter.

To investigate the protonation state of carboxyl groups in CHex-Dex at varying pH, acid-base titration of CHex-Dex was conducted (Figure 3). CHex-Dex changed their protonation states depending on pH in alkali and weakly acidic pH regions. Compared with CHex40-Dex, CHex-Dex having 57%, 73% and 86% of CHex groups showed higher pKa values (Table 3). This might result from the proximity effects between carboxyl groups on CHex-Dex as reported in previous literature [15]. As a comparison, pKa values of MGlu group-introduced dextran were listed in Table 3. In comparison with dextran derivatives having almost same carboxyl groups, CHex-Dex exhibited higher pKa than that of MGlu-Dex. In general, pKa of carboxyl groups in poly(carboxylic acid)s increased with increasing their hydrophobicity. Especially, hydrophobic structure next to carboxylate promotes the protonation of carboxyl groups, resulting in an increase of pKa [17,18,19,20,21,22]. Therefore, hydrophobic spacer unit in CHex groups promoted protonation of carboxyl groups, resulting in higher pKa than MGlu-Dex. These results are consistent with our previous literature using polyallylamine or poly(glycidol)s [18,21].

**Figure 4:**
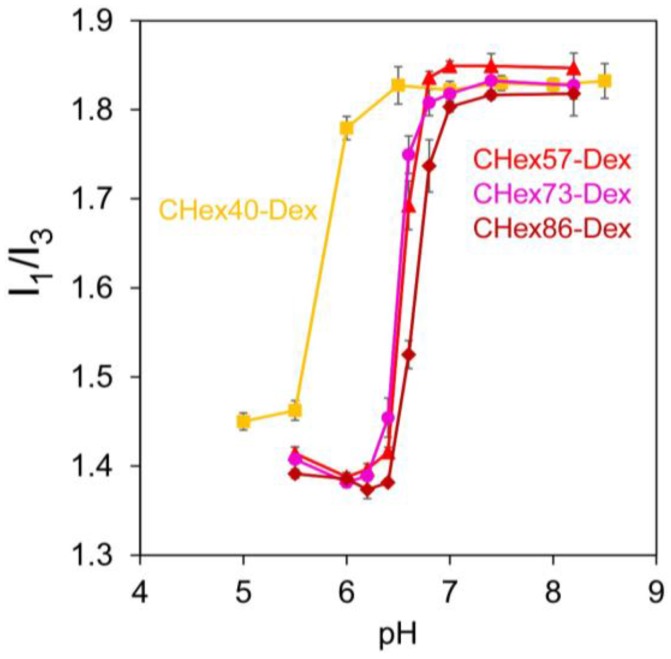
pH-Dependence of *I*_1_/*I*_3_ of pyrene fluorescence in the presence of dextran derivatives dissolving in 30 mM sodium acetate and 120 mM NaCl solution of varying pH. Concentrations of polymers and pyrene were 0.25 mg/mL and 0.6 μM, respectively. *I*_1_/*I*_3_ was defined as the fluorescence intensity ratio of the first band at 373 nm to the third band at 384 nm.

Protonation of carboxyl groups in poly(carboxylic acid)s induced coil-globule transition of polymer chains with formation of hydrophobic domains [19,20]. Next, the formation of hydrophobic domain of CHex-Dex was investigated using pyrene as a fluorescence probe. The pyrene fluorescence intensity ratio of the first (373 nm) to the third (384 nm) peaks, *I*_1_/*I*_3_, has been used to evaluate the micro-environmental polarity surrounding the pyrene molecule [18,21,22,23]. Figure 4 represents the *I*_1_/*I*_3_ ratio of pyrene fluorescence of CHex-Dex as a function of pH. At pH 8–7, the *I*_1_/*I*_3_ ratios of pyrene were around 1.8 for all dextran derivatives, indicating that these polymers formed no domain with a hydrophobic nature. In contrast, the *I*_1_/*I*_3_ ratio suddenly decreased below at pH 6.5 for CHex40-Dex or 7.0 for other CHex-Dex and reached to around 1.4. Considering the protonation curves (Figure 3), around 60% of carboxyl groups in CHex-Dex were protonated at these pH region. This indicates that CHex-Dex could form strong hydrophobic domains even though 40% of carboxyl groups in CHex-Dex still have anionic charges. According to the previous literature [15], the *I*_1_/*I*_3_ ratio for MGlu-Dex is around 1.7 even after most of carboxyl groups are protonated, which might reflect hydrophilic nature of dextran backbone. Therefore, hydrophobic interaction between protonated CHex units might mainly contribute the formation of hydrophobic domains because of their bulky and hydrophobic structure compared with MGlu units.

**Figure 5:**
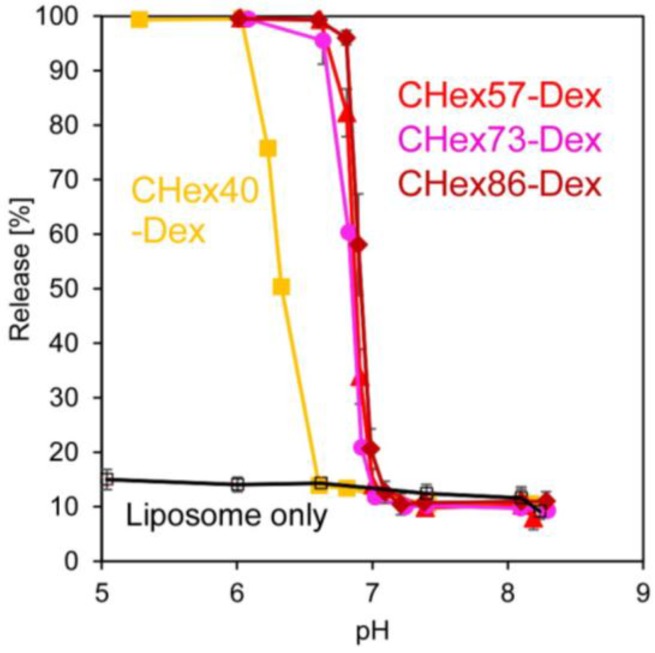
pH-Dependence of pyranine release from liposomes after addition of various dextran derivatives at various pH and 37 °C. Content release after 30 min-incubation is shown. Lipid concentration was 2.0 × 10^−5^ M. The ratio by weight of lipid: polymer is 9:1.

Next, interaction of dextran derivatives with lipid membrane was investigated. Fluorescence dye, pyranine-loaded egg yolk phosphatidylcholine (EYPC) liposomes were incubated with CHex-Dex at varying pH and release of pyranine was monitored. Figure S10 shows time courses of pyranine release from liposomes after addition of CHex-Dex. At alkali or neutral pH, addition of CHex-Dex hardly affected the leakage of pyranine from liposomes, whereas remarkable content release was observed within a few minutes at low pH. Figure 5 represents pH-dependence of pyranine release after 30 min-incubation. All CHex-Dex induced content release responding to pH decrease and pH region where content release occurred was quite narrow and totally corresponded to pH region where CHex-Dex formed hydrophobic domains (Figure 4). These results indicate that the destabilization of liposomal membrane by CHex-Dex with hydrophobic domains induced content release from liposomes. Thus, pH-responsive dextran derivatives which could destabilize lipid membrane effectively were successfully developed.

### 2.2. Preparation of Dextran Derivative-Modified Liposomes

So far, basic characteristics of dextran derivatives were evaluated using dextran derivatives without C_10_ groups. Next, anchor-introduced polymers were used for surface modification of liposomes. Dextran derivative-modified liposomes were prepared by lipid-thin film hydration methods as previously reported [15]. Mixed thin film of EYPC and dextran derivatives having anchor moieties was dispersed in PBS containing OVA as a model antigenic protein. Then, resultant lipid/polymer suspension was passed through polycarbonate membranes with pore size of 100 nm and unloaded OVA was removed by repeated ultracentrifugation. Table 4 shows the size and zeta potential of resultant liposomes. All liposomes exhibited a size of about 100 nm, which corresponds to pore size of polycarbonate membrane for extrusion. Dextran derivative-modified liposomes possessed more negative zeta potentials than that of unmodified liposomes. This might reflect the modification of dextran derivatives having carboxyl groups on liposomal surface.

**Figure 6:**
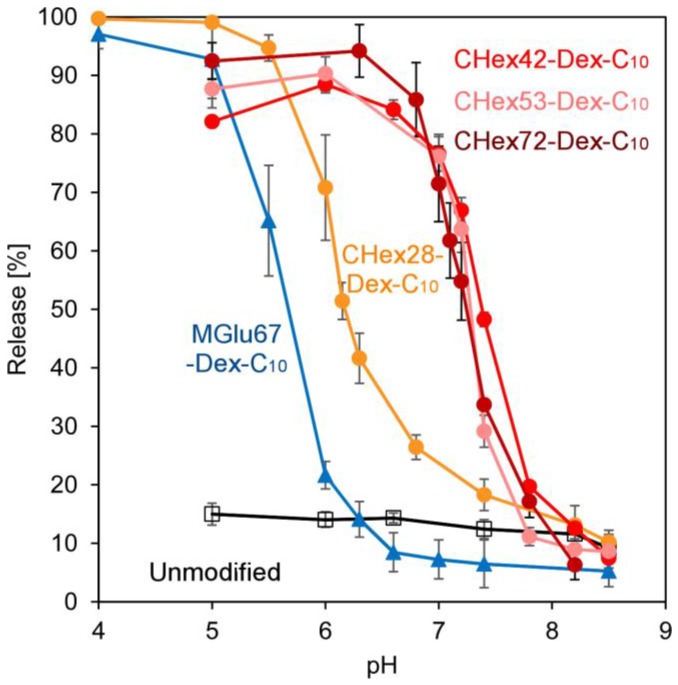
pH-Dependence of pyranine release from EYPC liposomes modified with or without 10 wt % CHex-Dex-C_10_ or 30 wt % MGlu-Dex-C_10_ at 37 °C after 30 min-incubation. Lipid concentrations were 2.0 × 10^−5^ M.

Next, pH-responsive content release from dextran derivative-modified liposomes was investigated. Pyranine were encapsulated in liposomes instead of OVA and pH-responsive release behaviors were evaluated (Figure S11 and Figure 6). In our previous literature, weight ratio of lipid: polymer was optimized as 7:3 for their pH-sensitivity. Actually, MGlu67-Dex-C_10_-modified liposomes with weight ratio of 7:3 exhibited pH-responsive release behavior at weakly acidic pH region (Figure 6). However, we could not obtain stable liposomes modified with CHex-Dex-C_10_ at weight ratio of 7:3. After optimization of lipid: polymer ratio in stability of liposomes, encapsulation efficiency of contents and evaluation of pH-sensitivity (Figure S12), lipid: polymer ratio was set to 9:1 for preparation of CHex-Dex-C_10_-modified liposomes. Resultant liposomes induced content release within a few minutes at extremely weakly acidic pH (Figure S11), suggesting that CHex-Dex became hydrophobic on liposome surface after protonation of carboxyl groups and liposomal membrane was immediately destabilized. CHex-Dex-C_10_-modified liposomes showed content release at higher pH region than MGlu-Dex-C_10_ modified liposomes. In addition, CHex-Dex-C_10_ having high amount of CHex groups tends to induce content release at high pH region (Figure 6). These results might reflect the difference of pKa and hydrophobicity of CHex-Dex having various amounts of CHex groups and MGlu-Dex (Table 3 and Figure 4).

### 2.3. Interaction of Liposomes with Dendritic Cells

**Figure 7:**
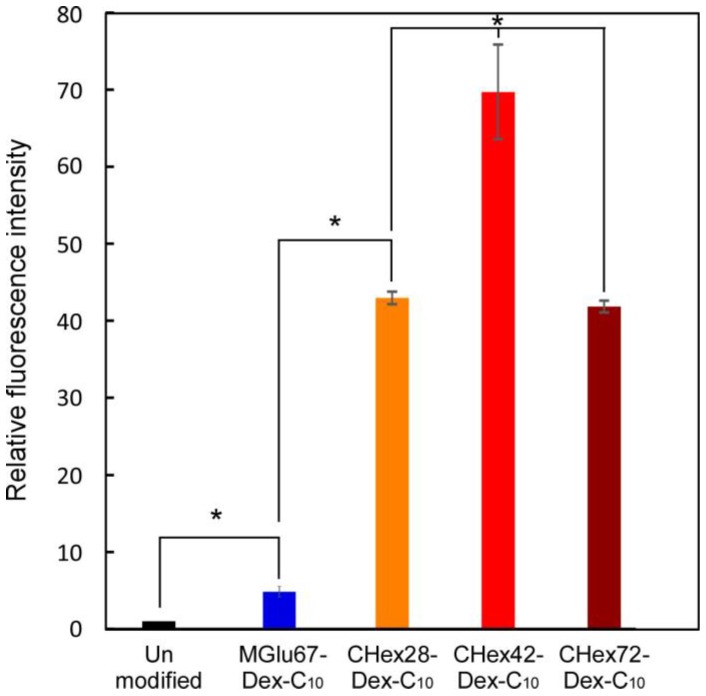
Relative fluorescence intensity for DC2.4 cells treated with DiI-labeled liposomes modified with or without 30 wt % MGlu67-Dex-C_10_ or 10 wt % CHex-Dex-C_10_ having various amounts of CHex groups. DC2.4 cells were incubated with liposomes for 4 h at 37 °C in serum-free medium. * *p* < 0.01.

Next, cellular association of liposomes with dendritic cells was examined to evaluate their antigen delivery performance. OVA-loaded liposomes were fluorescently labeled with fluorescence dye with hydrophobic moieties, DiI. These liposomes were applied to DC2.4 cells, a murine dendritic cell line, and fluorescence intensity of cells were measured using a flow cytometer (Figure 7). MGlu67-Dex-C_10_-modified liposomes showed five times higher cellular association than that of unmodified liposomes. Dendritic cells have scavenger receptors that recognize anionic molecules on aged cells or apoptotic cells [24,25,26,27]. Modification of liposomes with poly(carboxylic acid)s is known to increase cellular association of liposomes by APCs [28]. Our previous literatures also revealed that carboxylated poly(glycidol)- or carboxylated polysaccharide-modified liposomes were recognized by scavenger receptors and cellular association of liposomes increased [16,29]. Therefore, MGlu67-Dex-C_10_-modified liposomes might be also recognized by scavenger receptors on DC2.4 cells. Compared with MGlu67-Dex-C_10_, modification of CHex-Dex-C_10_ strongly increased cellular association of liposomes (Figure 7). Cellular association of CHex-Dex-C_10_-modified liposomes was 40–70 times higher than unmodified liposomes and over 10 times higher than MGlu67-Dex-C_10_-modified liposomes. As shown in Table 3, protonation state of MGlu-Dex at physiological pH is around 7%–22%, whereas those of CHex-Dex are 27%–49%. This might suggest that CHex-Dex on liposome surface possess more hydrophobic nature than MGlu-Dex, which increased the interactions with cells. Among CHex-Dex-C_10_ having various amounts of CHex groups, CHex42-Dex-C_10_ showed the highest cellular association (Figure 7). Compared with CHex42-Dex-C_10_, CHex28-Dex-C_10_ might have low protonation state, which might decrease the hydrophobic interaction with cells. CHex72-Dex-C_10_ has almost twice carboxylates, which might cause electrostatic repulsion with cellular surface and decrease cellular association of liposomes. Therefore, medium amounts of CHex group-introduced dextran derivatives exhibited the highest interaction with cells, which is consistent with our previous results in MGlu group-introduced polysaccharide derivatives [15,16].

**Figure 8:**
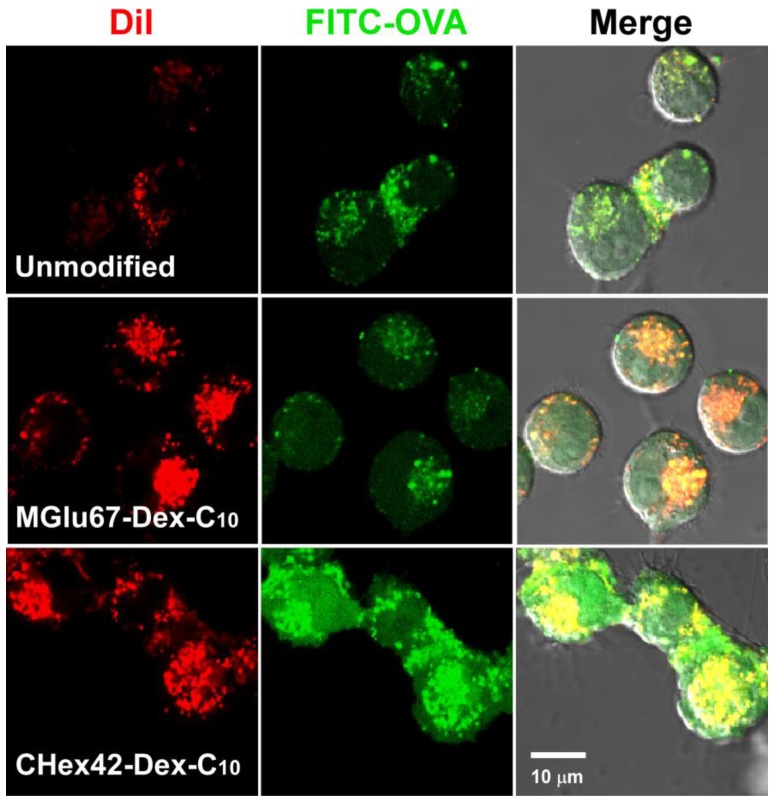
Confocal laser scanning microscopic (CLSM) images of DC2.4 cells treated with DiI-labeled and FITC-OVA-loaded EYPC liposomes modified with or without 30 wt % MGlu67-Dex-C_10_ or 10 wt % CHex42-Dex-C_10_ for 4 h at 37 °C in serum-free medium. Scale bar represents 10 μm. DiI-fluorescence intensity for liposome modified with CHex42-Dex-C_10_ was decreased to distinguish the distribution of liposomes. Lipid concentration was 5.0 × 10^−4^ M.

Intracellular delivery performance of antigenic proteins was further investigated using DiI-labeled and FITC-OVA-loaded liposomes (Figure 8). In the case of cells treated with unmodified liposomes, red and green fluorescence was observed as dots within cells. Considering liposomes are mainly internalized to cells via endocytosis, both liposomes and FITC-OVA molecules might be trapped in endosome/lysosome. For cells treated with MGlu67-Dex-C_10_-modified liposomes, DiI fluorescence was observed as dots but increased, reflecting increase of cellular association of liposomes (Figure 7). According to the result of co-staining using LysoTracker, DiI-fluorescence derived from liposomes were totally co-localized with LytoTracker fluorescence, indicating that liposomes exist in endosome/lysosomes (Figures S13 and S14). In Figure 8 and Figure S13, green fluorescence dots were overlapped with red and/or blue fluorescence but green fluorescence was also observed from different site from red and/or blue fluorescence. This indicates that MGlu67-Dex-C_10_-modified liposomes induced destabilization of endosomal membrane responding to acidic pH in endosomes and released FITC-OVA molecules from endosomes. CHex42-Dex-C_10_-modified liposomes exhibited further strong intracellular delivery performance and strong green fluorescence was observed from whole cells reflecting their hydrophobic nature and strong interaction with lipid membranes (Figure 4 and Figure 5). Because OVA contents per liposomes were almost identical in each liposome, the difference of FITC fluorescence directly reflect the difference of FITC-OVA amounts inside of cells (Figure S15). To investigate the effect of CHex group contents on cytoplasmic delivery performance, CHex28-Dex-C_10_-, CHex42-Dex-C_10_- and CHex72-Dex-C_10_-modified liposomes were applied to DC2.4 cells and observed by CLSM on same microscopy setting (Figure S16). Reflecting the results of cellular association, CHex42-Dex-C_10_-modified liposomes delivered FITC-OVA molecules into cytosol more efficiently than other CHex-Dex-C_10_-modified liposomes. This was also confirmed from the decrease of colocalization of FITC fluorescence with DiI fluorescence (Figure S17). These results indicate that both carboxylated unit contents and their spacer structures are important to obtain liposomes with high intracellular delivery performance to dendritic cells. Cytoplasmic delivery of antigenic proteins induces MHC class I-restricted antigen presentation. Actually, MGlu-Dex-C_10_-modified liposomes induced MHC class I-restricted antigen presentation and antigen-specific cellular immune responses [15]. Therefore, CHex-Dex-C_10_-modified liposomes are expected to induce MHC class I-restricted antigen presentation and stronger cellular immunity than MGlu-Dex-C_10_-modified liposomes.

### 2.4. Activation of Dendritic Cells by Dextran Derivatives

**Figure 9:**
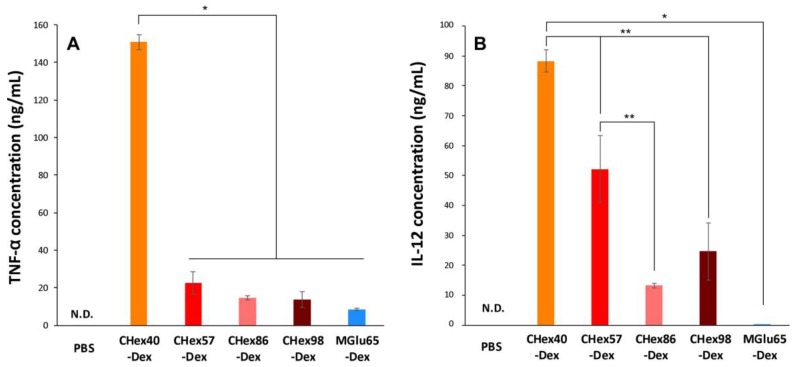
TNF-α (**A**) and IL-12 (**B**) production from DC2.4 cells treated with liposomes modified with dextran derivatives (1 mg/mL) for 24 h. ** *p* < 0.05. * *p* < 0.01.

For induction of cellular immunity, not only cytoplasmic delivery of antigen but also activation of dendritic cells are necessary. According to our previous results, the introduction of MGlu groups to dextran, curdlan or mannan promoted their cytokine production [16]. Therefore, the activation properties of dendritic cells by CHex-Dex were evaluated. Here, CHex-Dex without anchor groups was used because anchor groups are buried in the lipid membrane of polymer-modified liposomes and CHex-Dex on the liposome surface might be recognized by dendritic cells. DC2.4 cells were cultured in the presence of dextran derivatives for 24 h and the production of Th1 cytokines (TNF-α and IL-12) was measured by ELISA (Figure 9). As presented in Figure 9, TNF-α and IL-12 production from the DC2.4 cells was observed by treatment of MGlu65-Dex, which is consistent with our previous results [16]. Surprisingly, CHex40-Dex induced eighteen times higher TNF-α production and over 600 times higher IL-12 production from dendritic cells than those of MGlu65-Dex. These results indicate that the introduction of hydrophobic CHex groups is quite effective for activation of dendritic cells. Although further introduction of CHex groups to dextran decreased the cytokine production, which is the opposite result for the case of MGlu-Dex [16], CHex-Dex with high CHex group contents still showed over 100 times higher IL-12 production than MGlu65-Dex. Activation of APCs by polysaccharide derivatives having carboxyl groups or sulfate groups has been reported [30,31]. But these activation properties are 2–3 times higher than parent polysaccharides. According to our previous report, MGlu65-Dex induced 2 times higher IL-12 production than parent dextran probably because of high density of carboxyl groups on polysaccharide chain [16]. In this study, CHex40-Dex induced over 600 times higher IL-12 production than MGlu65-Dex (Figure 9B). These results suggest that hydrophobicity of spacer groups are quite important for activation of dendritic cells rather than the density of carboxyl groups. To the best of our knowledge, this is the first report to reveal the effect of spacer groups of carboxylated polysaccharide derivatives on their adjuvant function. Moreover, IL-12 production from dendritic cells strongly activates cellular immunity [32]. Therefore, CHex-Dex-modified liposomes are expected to not only deliver antigen into cytosol but also activate antigen-specific cellular immunity. We are currently attempting the evaluation of the immunity-inducing properties in vivo and antitumor effects of CHex-Dex-modified liposomes to confirm their potency for cancer immunotherapy.

## 3. Materials and Methods

### 3.1. Materials

EYPC was kindly donated by NOF Co. (Tokyo, Japan). 1,2-Cyclohexanedicarboxylic anhydride, OVA, fluorescein isothiocyanate (FITC), *p*-xylene-bis-pyridinium bromide (DPX) were purchased from Sigma (St. Louis, MO, USA). Dextran having molecular weight of 70,000, 1-aminodecane, pyranine and Triton X-100 were obtained from Tokyo Chemical Industries Ltd. (Tokyo, Japan). 4-(4,6-Dimethoxy-1,3,5-triazin-2-yl)-4-methyl morpholinium chloride (DMT-MM) was from Wako Pure Chemical Industries Ltd. (Osaka, Japan). 1,1′-Dioctadecyl-3,3,3′,3′-tetramethylindocarbocyanine perchlorate (DiI) was from Life Technologies. Dialysis tube (molecular weight cutoff: 10 kDa) was purchased from Sekisui Medical Co., Ltd. (Tokyo, Japan). FITC-OVA was prepared by reacting OVA (10 mg) with FITC (11.8 mg) in 0.5 M NaHCO_3_ (4 mL, pH 9.0) at 4 °C for three days and subsequent dialysis [13]. 3-Methyl glutarylated dextrans (MGlu65-Dex and MGlu67-Dex-C_10_) were prepared as previously reported [15].

### 3.2. Synthesis of Dextran Derivatives

2-Carboxycyclohexane-1-carboxylated dextran (CHex-Dex) was prepared by reaction of dextran with 1,2-cyclohexanedicarboxylic anhydride. Experimental conditions were shown in Table 1. A given amount of dextran and LiCl were dissolved in *N*,*N*-dimethylformamide (DMF) and 1,2-cyclohexanedicarboxylic anhydride (CHex anhydride) was added to the solution. The mixed solution was kept at various temperatures for 24 h with stirring under argon atmosphere. Then, the reaction mixture was evaporated and saturated sodium hydrogen carbonate aqueous solution was added to the reaction mixture for neutralization and dialyzed against water for 5 days. The product was recovered by freeze-drying. ^1^H NMR for CHex-Dex (400 MHz, D_2_O + NaOD, Figures S1–S5): δ 1.3–2.0 (m, −cyclo−C*H*_2_), 2.6 (m, cyclo−C*H*), 3.4–4.1 (br, glucose 2*H*, 3*H*, 4*H*, 5*H*, 6*H*), 5.0 (br, glucose 1*H*).

As anchor moieties for fixation of CHex-Dex onto liposome membranes, 1-aminodecane was combined with carboxyl groups of CHex-Dex. Experimental conditions were shown in Table 2. Each polymer was dissolved in water around pH 7.4, and 1-aminodecane (0.1 equiv. to hydroxyl group of polymer) was reacted to carboxyl groups of the polymer using DMT-MM (0.1 equiv. to hydroxyl group of polymer) at room temperature for 5–36 h with stirring. The obtained polymers were purified by dialysis in water. The compositions for polymers were estimated using ^1^H NMR. ^1^H NMR for CHex-Dex-C_10_ (400 MHz, D_2_O + NaOD, Figures S6–S9):δ 0.9 (br, −CO−NH−CH_2_−(CH_2_)_8_−C*H*_3_), 1.2–2.0 (m, −cyclo−C*H*_2_, −CO−NH−CH_2_−(C*H*_2_)_8_−CH_3_), 2.6 (m, cyclo−C*H*), 3.2 (br, −CO−NH−C*H*_2_−(CH_2_)_8_−CH_3_), 3.4–4.1 (br, glucose 2*H*, 3*H*, 4*H*, 5*H*, 6*H*), 5.0 (br, glucose 1*H*).

### 3.3. Titration

To 50 mL of an aqueous solution of each polymer (carboxylate concentration: 1.2 × 10^−4^ M) was added an appropriate amount of 0.1 M NaOH solution to make pH 11.3. The titration was carried out by the stepwise addition of 0.01 M HCl at 4 °C and conductivity and pH of the solution were monitored.

### 3.4. Pyrene Fluorescence

A given amount of pyrene in acetone solution was added to an empty flask, and acetone was removed under vacuum. Polymer (0.25 mg/mL) dissolving in 30 mM sodium acetate and 120 mM NaCl solution of a given pH was added to the flask, yielding 0.6 μM concentration of pyrene. The sample solution was stirred overnight at room temperature, and emission spectra with excitation at 337 nm were recorded. The fluorescence intensity ratio of the first band at 373 nm to the third band at 384 nm (*I*_1_/*I*_3_) was analyzed as a function of solution pH.

### 3.5. Preparation of Liposomes

To a dry, thin membrane of EYPC (10 mg) was added 1.0 mL of OVA/PBS solution (pH 8.2, 4 mg/mL), and the mixture was vortexed at 4 °C. The liposome suspension was further hydrated by freezing and thawing, and was extruded through a polycarbonate membrane with a pore size of 100 nm. The liposome suspension was centrifuged with the speed of 55,000 rpm for 2 h at 4 °C twice to remove free OVA from the OVA-loaded liposomes. Polymer-modified liposomes were also prepared according to the above procedure using dry membrane of a lipid mixture with polymers (lipids/polymer = 9/1, *w*/*w* for CHex-Dex-C_10_ and 7/3, *w*/*w* for MGlu-Dex-C_10_).

### 3.6. Dynamic Light Scattering and Zeta Potential

Diameters and zeta potentials of the liposomes (0.1 mM of lipid concentration, pH 7.4) were measured using a Zetasizer Nano ZS ZEN3600 (Malvern Instruments Ltd., Worcestershire, UK). Data was obtained as an average of more than three measurements on different samples.

### 3.7. Release of Pyranine from Liposome

Pyranine-loaded liposomes were prepared as described above except that mixtures of polymers and EYPC were dispersed in aqueous 35 mM pyranine, 50 mM DPX, and 25 mM phosphate solution (pH 8.2). For the study of the interaction of polymers with lipid membranes, a given amount of the polymer dissolved in the same buffer (final concentration: 1.78 μg/mL) at 25 °C was added to a suspension of pyranine-loaded liposomes (lipid concentration: 2.0 × 10^−5^ M) in PBS of varying pH, and fluorescence intensity (512 nm) of the mixed suspension was followed with excitation at 416 nm using a spectrofluorometer (Jasco FP-6500, Tokyo, Japan). For the study of the release behavior of polymer-modified liposomes, polymer-modified liposomes encapsulating pyranine were added to PBS of varying pHs at 37 °C and fluorescence intensity of the suspension was monitored (lipid concentration: 2.0 × 10^−5^ M). The percent release of pyranine from liposomes was defined as:

Release (%) = (*F*_t_ − *F*_i_)/(*F*_f_ − *F*_i_) × 100

where *F*_i_ and *F*_t_ mean the initial and intermediary fluorescence intensities of the liposome suspension, respectively. *F*_f_ is the fluorescent intensity of the liposome suspension after the addition of TritonX-100 (final concentration: 0.1%).

### 3.8. Cell Culture

DC2.4 cell, which is an immature murine DC line, was provided from Kenneth L. Rock (University of Massachusetts Medical School, Worcester, MA, USA) and were grown in RPMI-1640 supplemented with 10% FBS (MP Biomedical, Inc., Santa Ana, CA, USA), 2 mM L-glutamine, 100 mM nonessential amino acid, 50 μM 2-mercaptoethanol (2-ME, Gibco) and antibiotics at 37 °C [33].

### 3.9. Cellular Association of Liposomes

Liposomes containing DiI were prepared as described above except that a mixture of polymer and lipid containing DiI (0.1 mol%) was dispersed in PBS. DC2.4 cells (1.5 × 10^5^ cells) cultured for 2 days in 12-well plates were washed with Hank’s balanced salt solution (HBSS), and then incubated in serum-free RPMI medium (0.5 mL). The DiI-labeled liposomes (1 mM lipid concentration, 0.5 mL) were added gently to the cells and incubated for 4 h at 37 °C. After the incubation, the cells were washed with HBSS three times. Fluorescence intensity of these cells was determined by a flow cytometric analysis (Cyto FLEX, Beckman Coulter, Inc., Brea, CA, USA). DiI fluorescence of each liposome was measured and the cellular fluorescence shown in Figure 7 was corrected using liposomal fluorescence intensity.

### 3.10. Intracellular Behavior of Liposomes

The FITC-OVA-loaded liposomes containing DiI were prepared as described above except that a mixture of polymer and lipid containing DiI (0.1 mol %) was dispersed in PBS containing FITC-OVA (4 mg/mL). DC2.4 cells (1.5 × 10^5^ cells) cultured for 2 days in 35-mm glass-bottom dishes were washed with HBSS, and then incubated in serum-free RPMI medium (1 mL). The FITC-OVA-loaded liposomes (1 mM lipid concentration, 1 mL) were added gently to the cells and incubated for 4 h at 37 °C. After the incubation, the cells were washed with HBSS three times. Confocal laser scanning microscopic (CLSM) analysis of these cells was performed using LSM 5 EXCITER (Carl Zeiss Co. Ltd., Oberkochen, Germany).

### 3.11. Cytokine Production from Cells Treated with Dextran Derivatives

The DC2.4 cells (5 × 10^4^ cells) cultured for 2 days in 12-well plates were washed with HBSS, and then incubated in serum-free RPMI medium (0.5 mL). Dextran derivatives (2 mg/mL, 0.5 mL) were added gently to the cells and incubated for 24 h at 37 °C. After the incubation, supernatants of cultured cells were collected for measurements of TNF-α and IL-12 using an enzyme-linked immunosorbent assay kit (ELISA Development Kit, PeproTech EC Ltd., London, UK) according to the manufacture’s instruction.

### 3.12. Statistical Analysis

Tukey-Kramer method was employed in the statistical evaluation of the results in Figure 7 and Figure 9.

## 4. Conclusions

In this study, carboxylated dextran derivatives having hydrophobic spacer groups were developed. CHex-Dex formed strong hydrophobic domains with pH decreasing and destabilized liposomal membrane immediately. These polymer-modified liposomes also showed contents release at low pH and efficient intracellular delivery performance compared with conventional dextran derivatives (MGlu-Dex). CHex-Dex also exhibited several hundred times higher IL-12 production from dendritic cells than MGlu-Dex. Therefore, CHex-Dex-modified liposomes are promising as antigen carriers with both intracellular delivery function and strong adjuvant functions to activate cellular immunity.

## Figures and Tables

**Table 1:** Synthesis of CHex-Dex.

Polymer	Dextran (g)	LiCl (g)	DMF (mL)	CHex-Anhydride (g)	Reaction Temperature (°C)	Yield (g)	Yield (%)	CHex (%) ^1^
CHex40-Dex	0.508	0.505	18	2.507	75	0.981	90	40
CHex57-Dex	0.998	0.985	12	5.016	90	1.129	43	57
CHex73-Dex	1.500	1.501	29	14.999	75	3.250	70	73
CHex86-Dex	1.170	0.986	16	10.290	100	3.530	87	86
CHex98-Dex	0.900	0.908	16	9.007	90	2.317	67	98

^1^ Determined by ^1^H NMR.

**Table 2:** Synthesis of CHex-Dex-C_10_.

Polymer	CHex-Dex (g)	n-Decyl Amine (mg)	DMT-MM (mg)	Yield (g)	Yield (%)	CHex (%) ^1^	Anchor (%) ^1^
CHex28-Dex-C_10_	0.507	54	107	0.430	90	28	4
CHex42-Dex-C_10_	0.816	86	174	0.794	95	51	6
CHex53-Dex-C_10_	0.500	46	81	0.426	96	53	4
CHex72-Dex-C_10_	1.214	95	68	0.532	43	76	5

^1^ Determined by ^1^H NMR.

**Table 3:** pKa and protonation degree at pH 7.4 of dextran derivatives.

Polymer	p*K*a	Protonation Degree at pH 7.4
CHex40-Dex	6.42	0.27
CHex57-Dex	7.24	0.46
CHex73-Dex	7.25	0.46
CHex86-Dex	7.34	0.49
MGlu13-Dex	5.30 ^1^	0.08
MGlu48-Dex	5.81 ^1^	0.07
MGlu76-Dex	6.63 ^1^	0.22

^1^ Reproduced from Ref. [15].

**Table 4:** Characteristics of liposomes.

Liposome	Size (nm)	Zeta Potential (mV)
Unmodified	135 ± 3	−10 ± 5
CHex28-Dex-C_10_	118 ± 4	−36 ± 0.2
CHex42-Dex-C_10_	120 ± 7	−31 ± 1
CHex53-Dex-C_10_	112 ± 1	−27 ± 1
CHex72-Dex-C_10_	109 ± 1	−37 ± 1
MGlu67-Dex-C_10_	104 ± 1	−33 ± 1

## Supplementary Materials

The following are available online at www.mdpi.com/2077-0375/7/3/41/s1, Figures S1–S9: NMR spectra for dextran derivatives, Figure S10: Time course of pyranine release from liposomes after addition of CHex-Dex, Figure S11: Time course of pyranine release from liposomes modified with dextran derivatives, Figure S12: Effect of polymer/lipid ratio on pH-sensitivity of CHex-Dex-C_10_-modified liposomes, Figures S13 and S14: Co-staining of end/lysosomes for liposome-treated DC2.4 cells, Figure S15: OVA amounts in liposomes, Figure S16: CLSM images of DC2.4 cells treated with CHex-Dex-C_10_-modified liposomes, Figure S17: Analysis of colocalization for FITC and DiI.
