# Supplementary material for: Development of pH-sensitive Dextran Derivatives with Strong Adjuvant Function and Their Application to Antigen Delivery
Source: Membranes (Basel). 2017 Aug 4;7(3):41. doi: 10.3390/membranes7030041 (PMC5618126; doi:10.3390/membranes7030041)
Supplement: Supplementary file 1 [file membranes-07-00041-s001.pdf]

Supplementary Materials

Development of pH-sensitive dextran derivatives with strong adjuvant function and their application to antigen delivery

Eiji Yuba*, Shinya Uesugi, Maiko Miyazaki, Yuna Kado, Atsushi Harada, and Kenji Kono

Department of Applied Chemistry, Graduate School of Engineering, Osaka Prefecture University, 1-1 Gakuen-cho, Naka-ku, Sakai, Osaka 599-8531, Japan

***Corresponding author: Eiji Yuba**
Tel: +81-722-54-9913; Fax: +81-722-54-9330; yuba@chem.osakafu-u.ac.jp

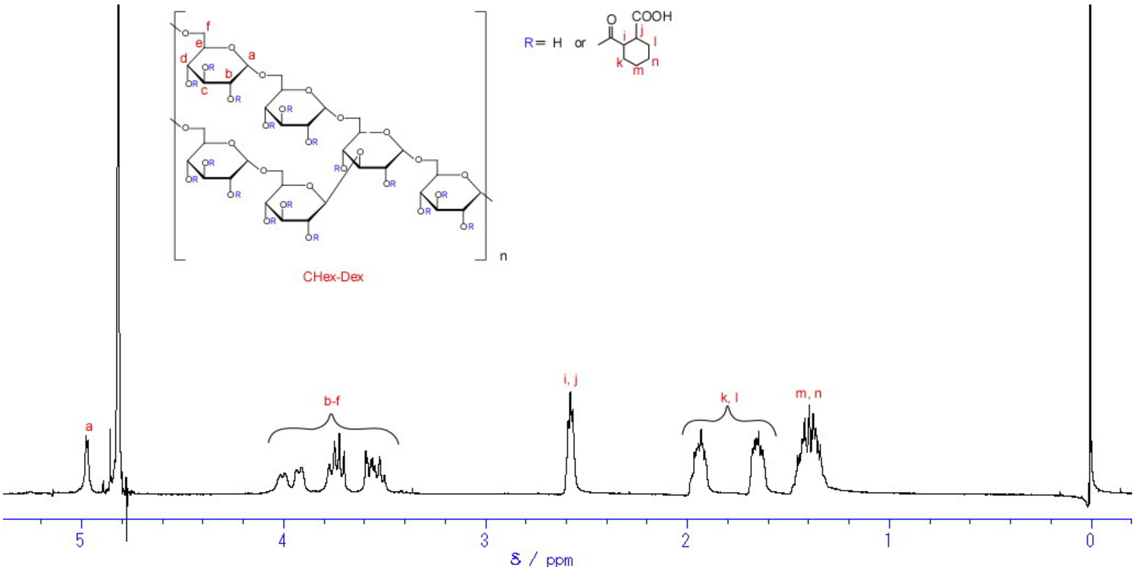

Figure S1. ^{1}H NMR chart of CHex40-Dex (400 MHz, D_2O+NaOD).

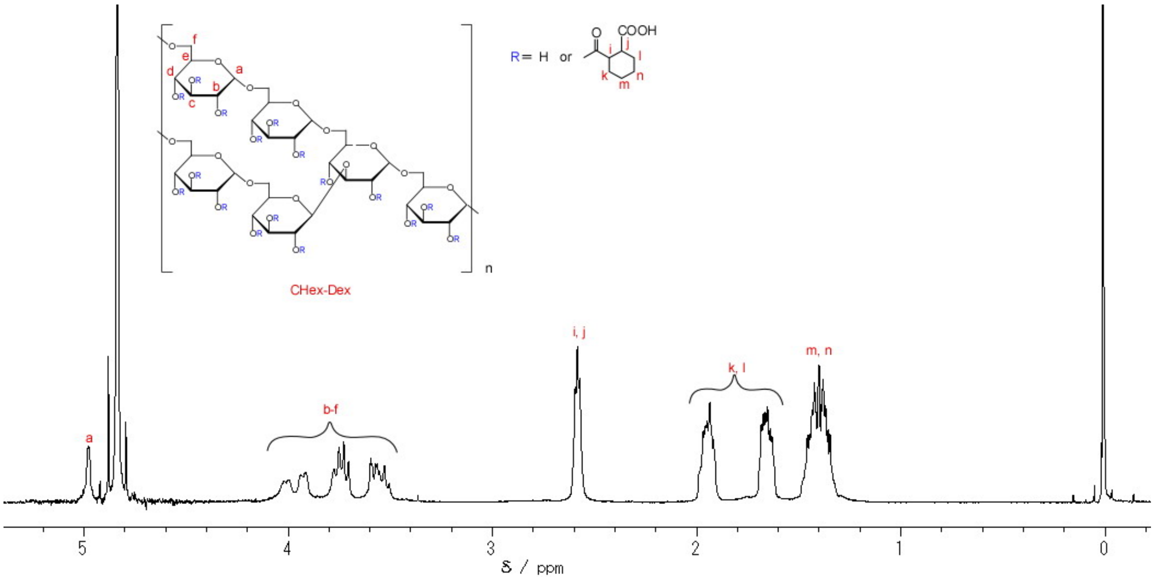

Figure S2. ^{1}H NMR chart of CHex57-Dex (400 MHz, D_2O+NaOD).

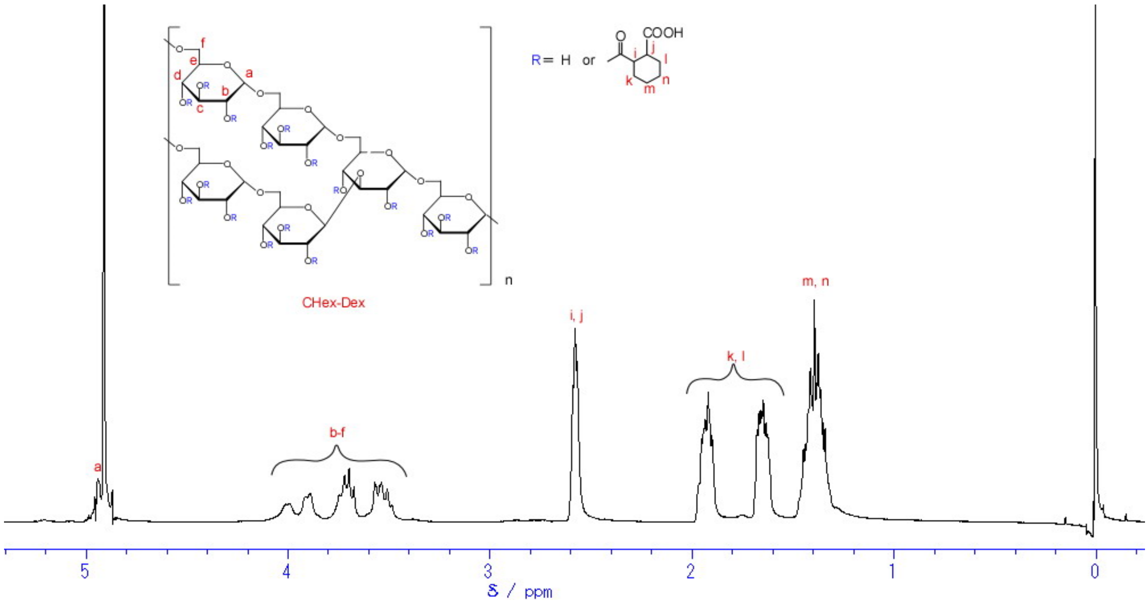

Figure S3. ^{1}H NMR chart of CHex73-Dex (400 MHz, D_2O+NaOD).

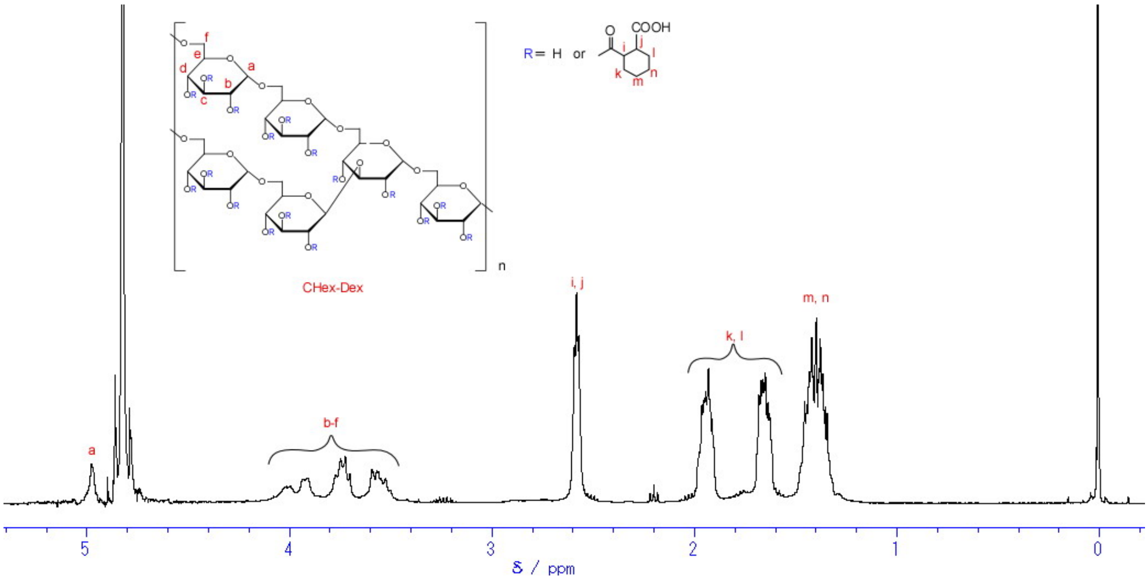

Figure S4. ^{1}H NMR chart of CHex86-Dex (400 MHz, D_2O+NaOD).

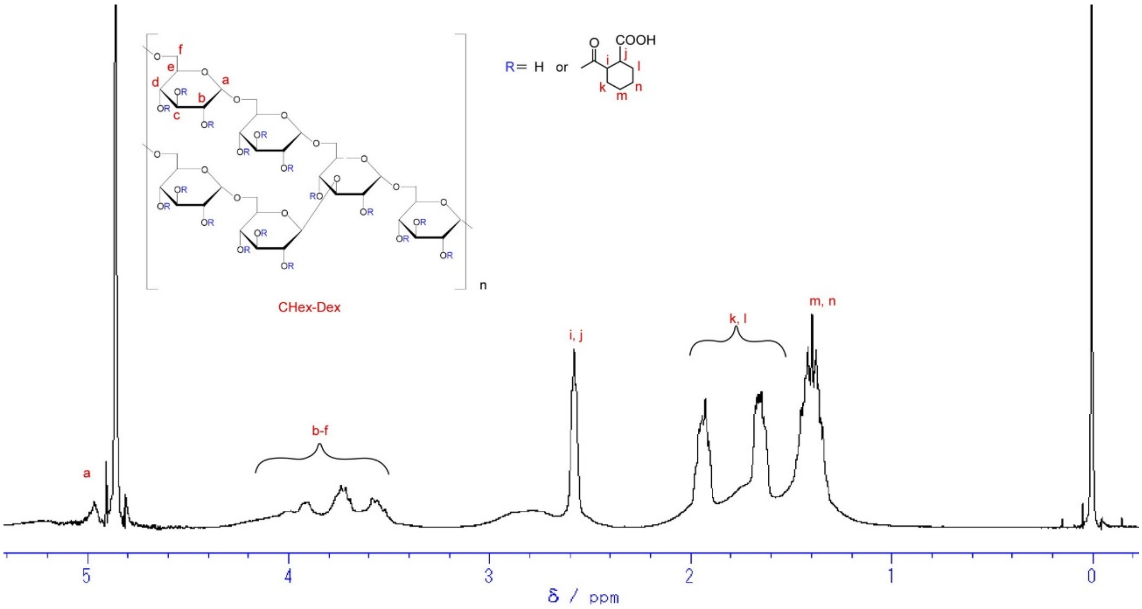

Figure S5. ^{1}H NMR chart of CHex98-Dex (400 MHz, D_2O+NaOD).

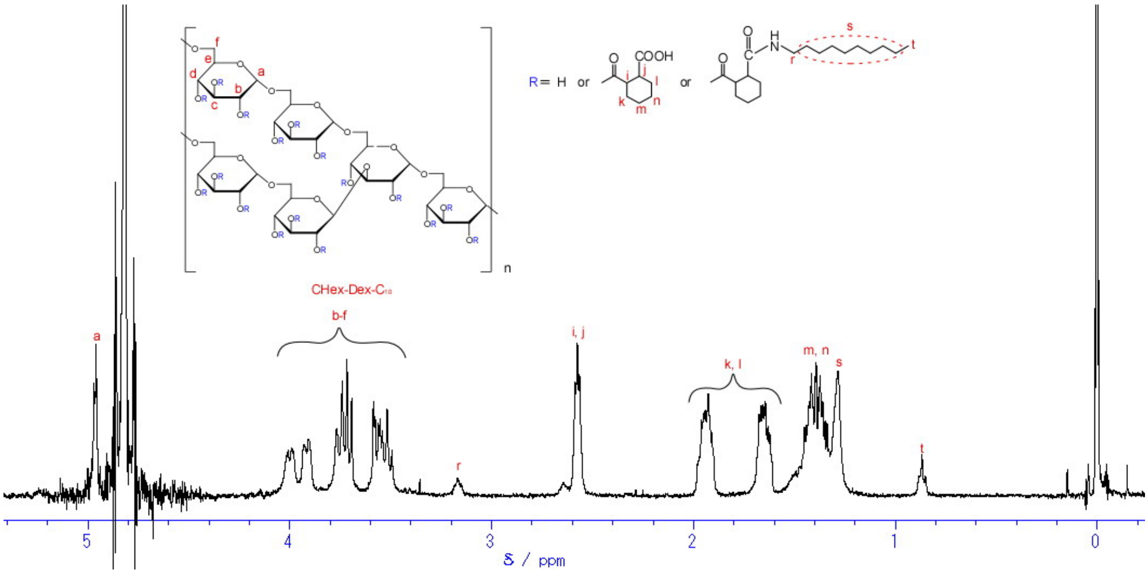

Figure S6. ^{1}H NMR chart of CHex28-Dex-C_{10} (400 MHz, D_2O+NaOD).

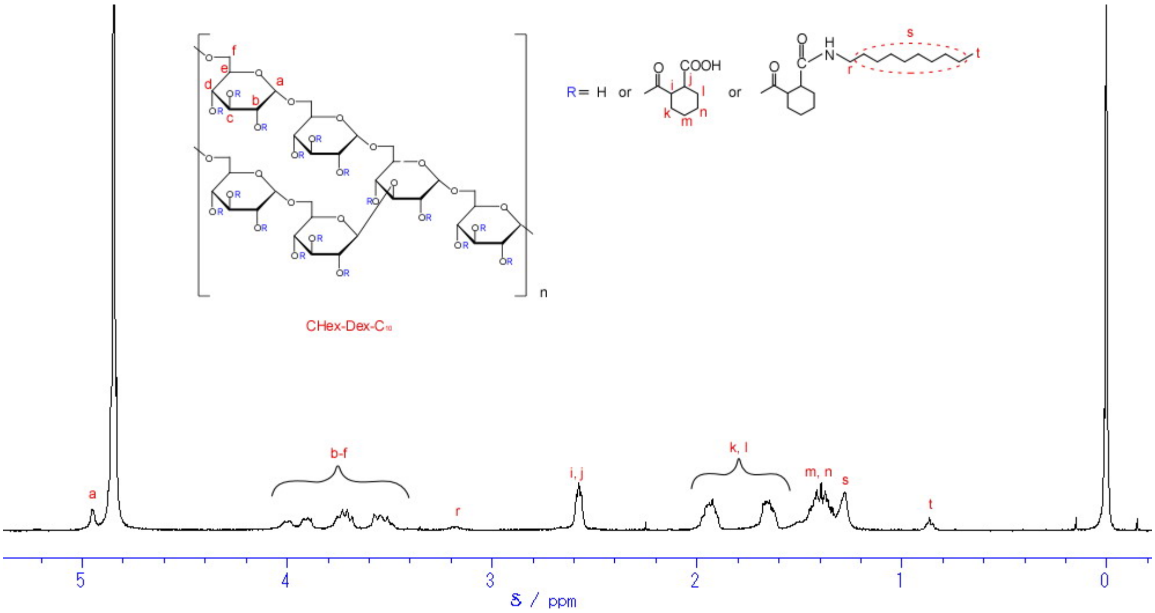

Figure S7. ^{1}H NMR chart of CHex42-Dex-C_{10} (400 MHz, D_2O+NaOD).

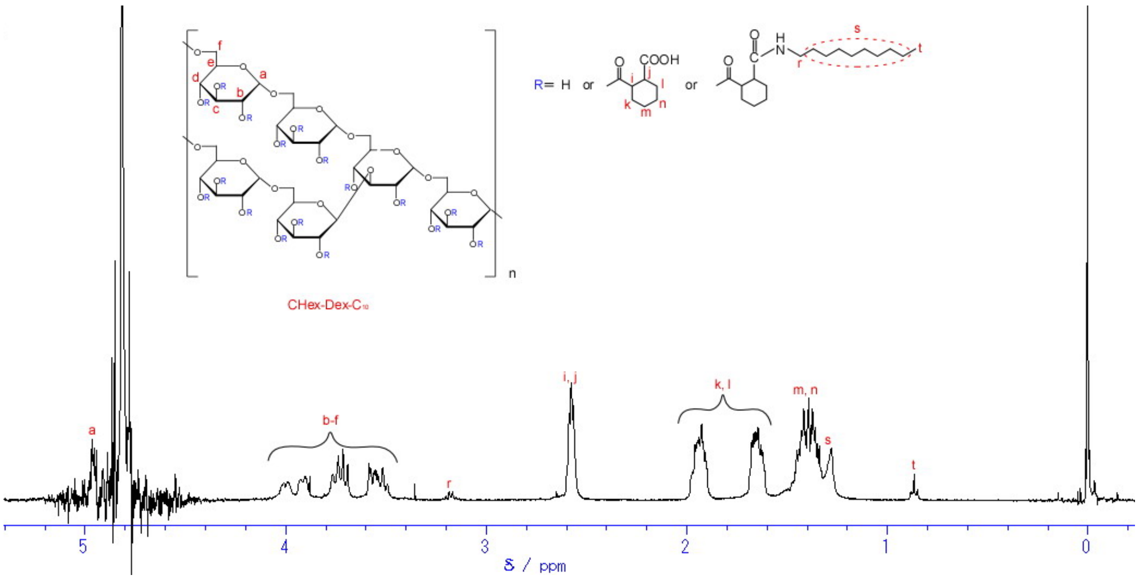

Figure S8. ^{1}H NMR chart of CHex53-Dex-C_{10} (400 MHz, D_2O+NaOD).

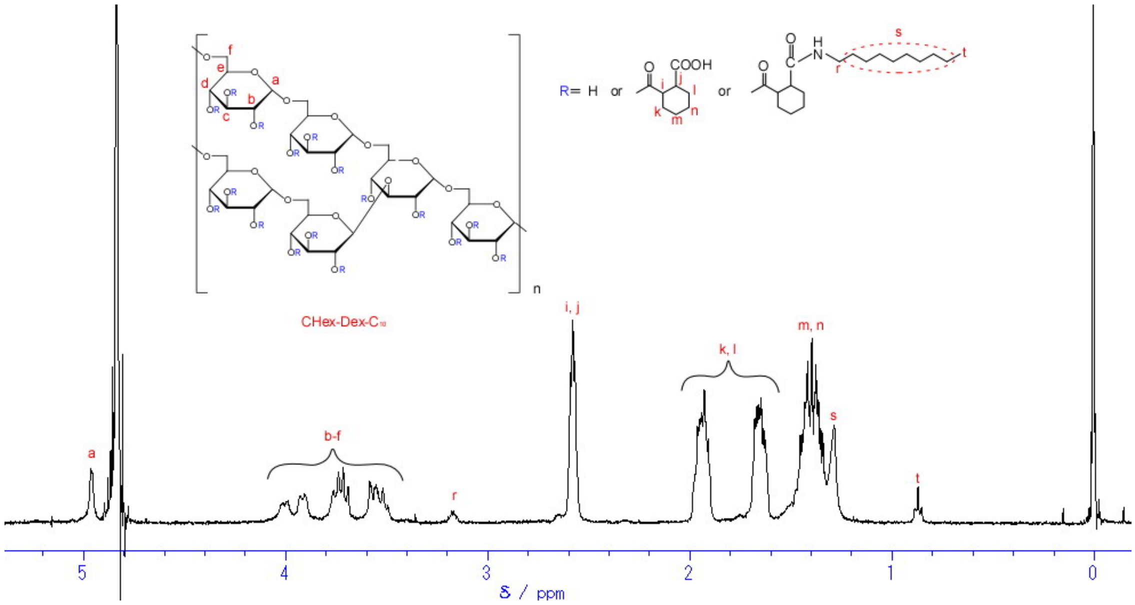

Figure S9. ^{1}H NMR chart of CHex72-Dex-C_{10} (400 MHz, D_2O+NaOD).

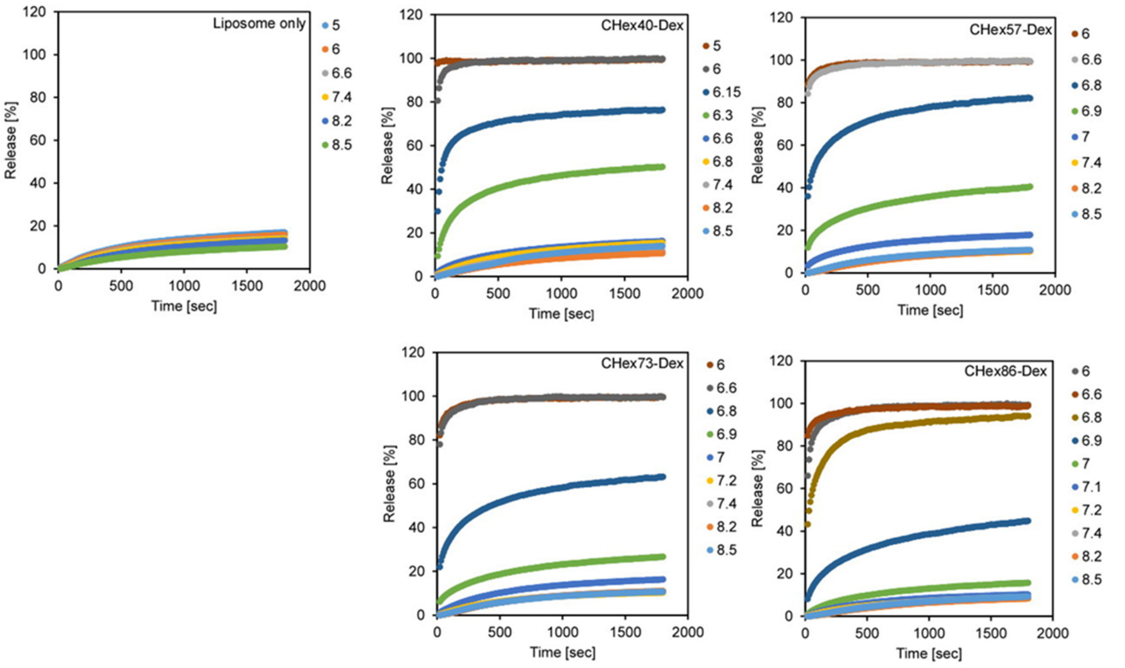

Figure S10. Time courses of pyranine release from EYPC liposomes at various pH after addition of various CHex-Dex. Lipid concentration was 2.0 × 10^{-5} M. The ratio by weight of lipid to polymer is 9 to 1. Measurements were performed in PBS solution at 37 °C.

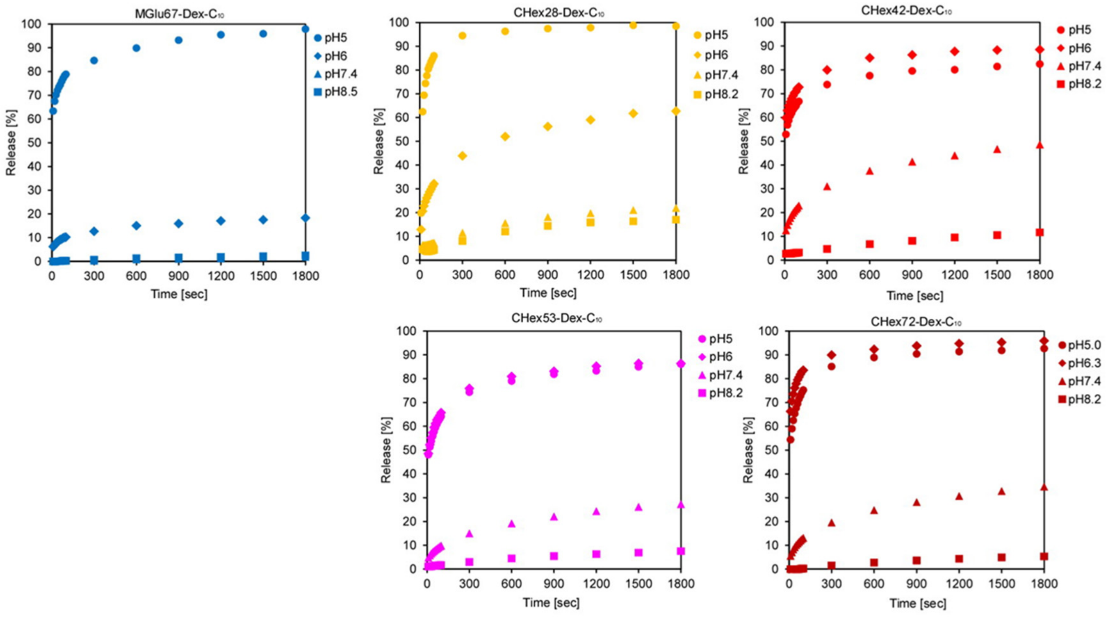

Figure S11. Time courses of pyranine release from EYPC liposomes modified with or without 10 wt% CHex-Dex-C_{10} or 30 wt% MGlu67-Dex-C_{10} at 37 °C. Lipid concentrations were 2.0×10^{-5} M.

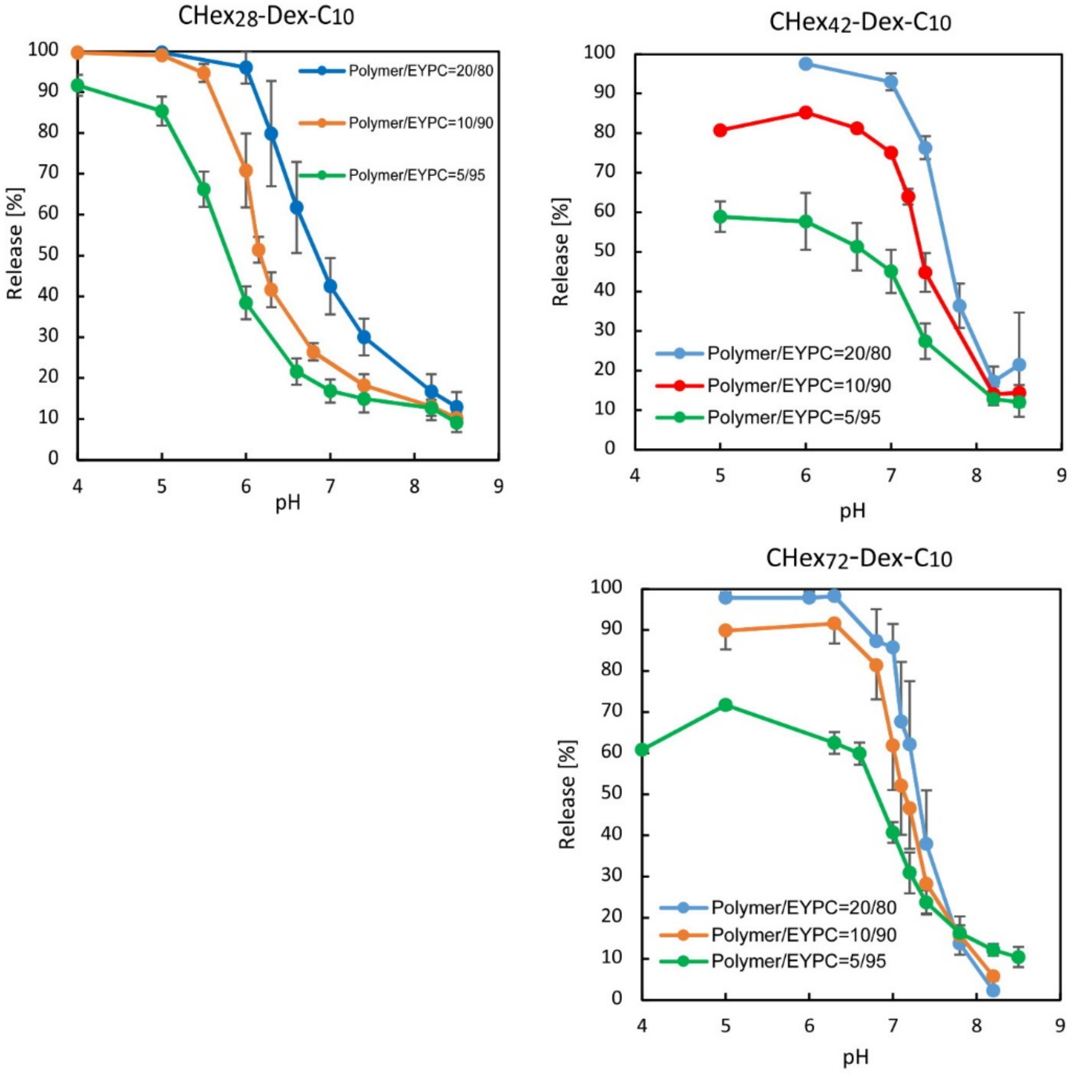

Figure S12. Effect of polymer/lipid ratio on pH-sensitivity of CHex-Dex-C_{10}-modified liposomes. Pyranine release from EYPC liposomes modified with various amounts of CHex-Dex-C_{10} at 37 °C after 30 min-incubation was evaluated. Lipid concentrations were 2.0×10^{-5} M.

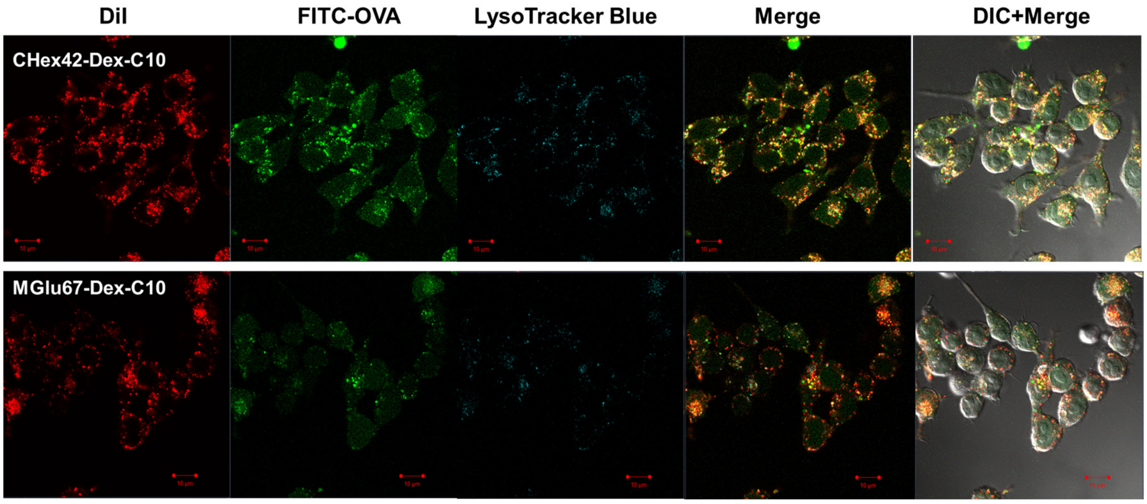

Figure S13. Confocal laser scanning microscopy (CLSM) images of DC2.4 cells treated with DiI-labeled and FITC-OVA-loaded EYPC liposomes modified with CHex42-Dex-C_{10} or MGlu67-Dex-C_{10} for 2 h at 37 °C in serum-free medium. Scale bar represents 10 μm. Lipid concentration was 5.0 × 10^{-4} M. Intracellular acidic compartments were stained using LysoTracker Blue.

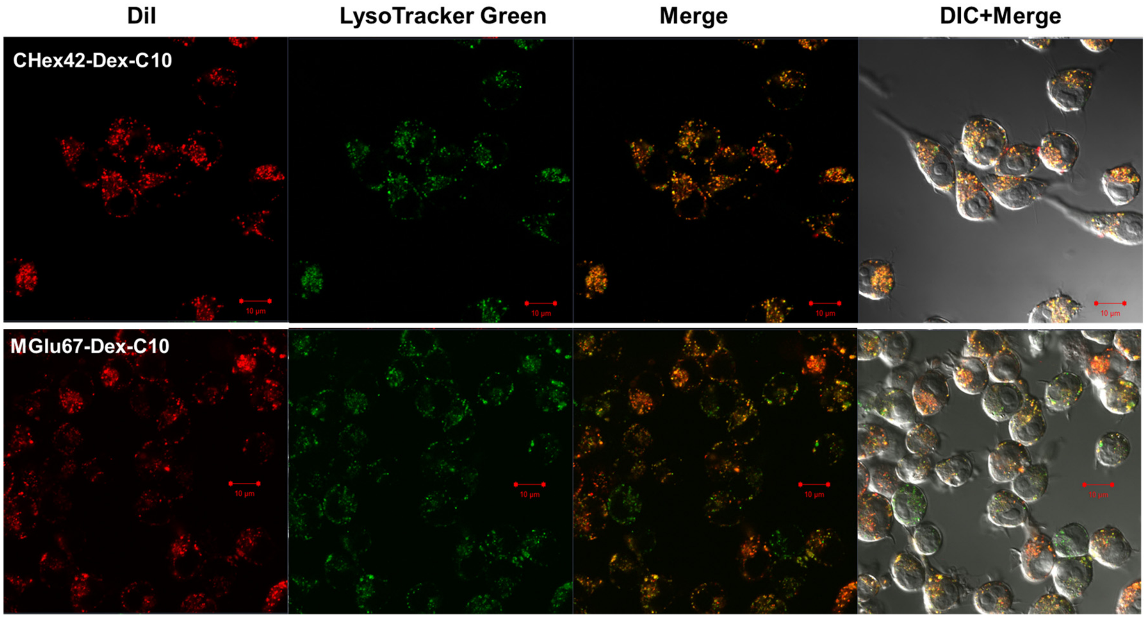

Figure S14. Most DiI fluorescence derived from liposomes co-localized with endo/lysosomes. CLSM images of DC2.4 cells treated with DiI-labeled EYPC liposomes modified with CHex42-Dex-C_{10} or MGlu67-Dex-C_{10} for 2 h at 37 °C in serum-free medium. Scale bar represents 10 μm. Lipid concentration was 5.0 × 10^{-4} M. Intracellular acidic compartments were stained using LysoTracker Green.

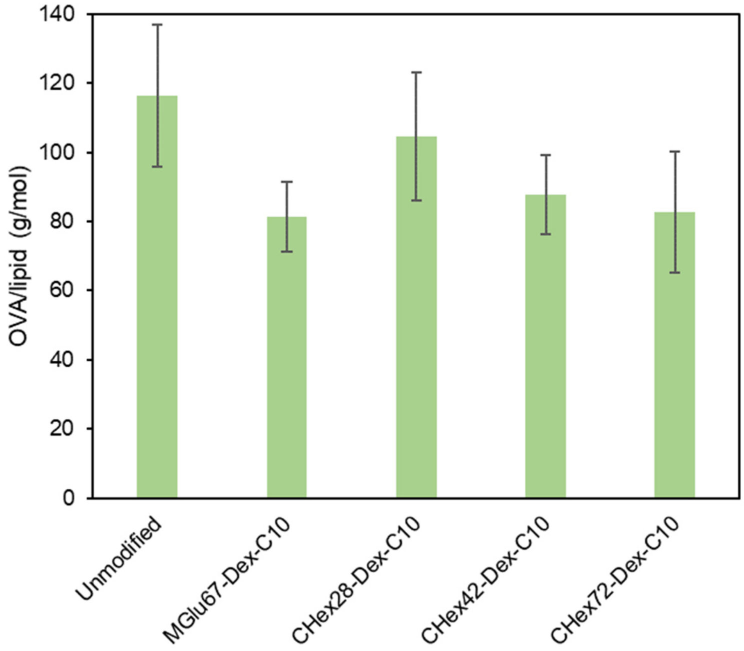

Figure S15. OVA amounts per lipid in various liposomes.

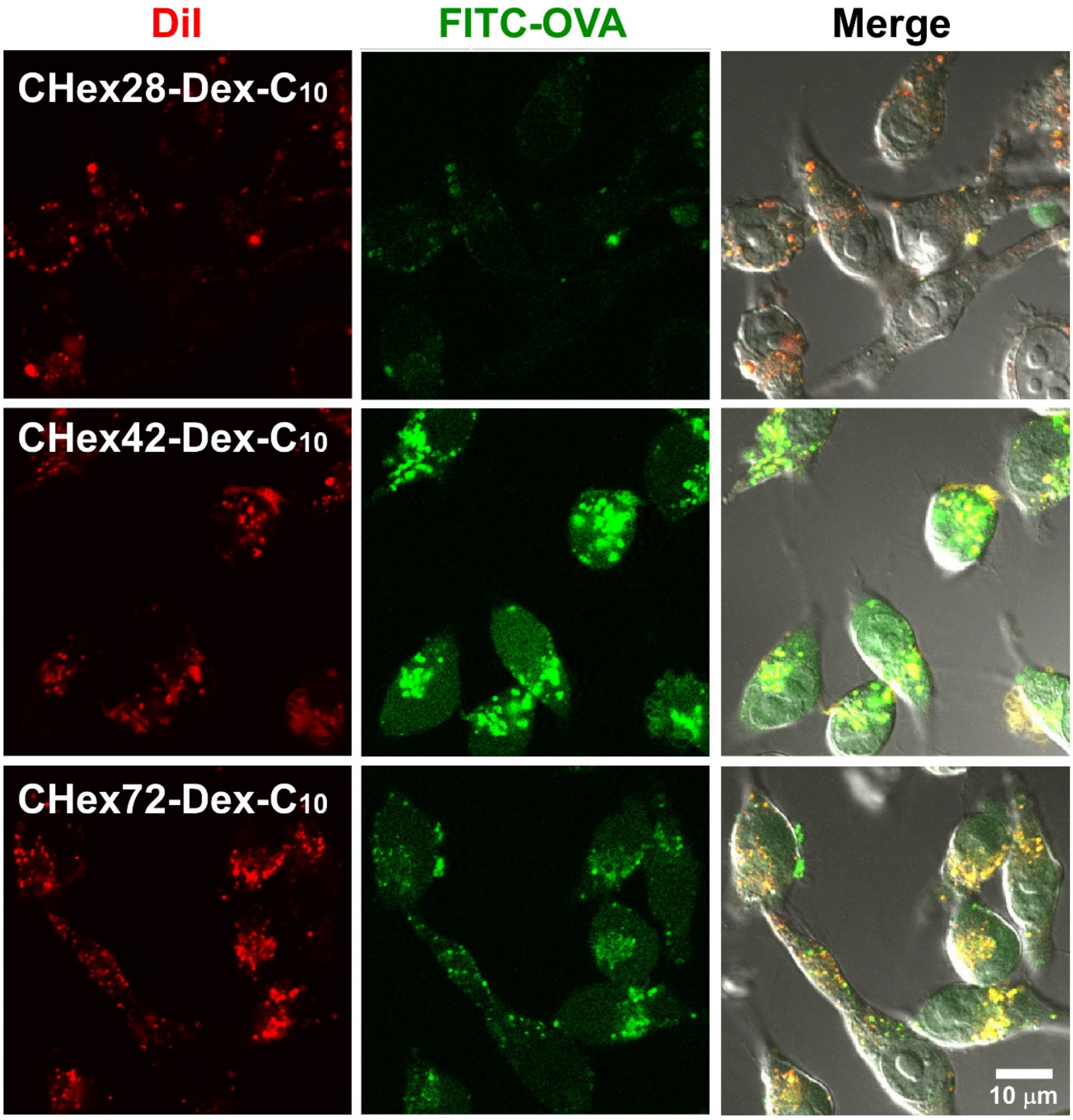

Figure S16. CLSM images of DC2.4 cells treated with DiI-labeled and FITC-OVA-loaded EYPC liposomes modified with CHex-Dex-C_{10} for 4 h at 37 °C in serum-free medium. Scale bar represents 10 μm. Lipid concentration was 5.0 × 10^{-4} M.

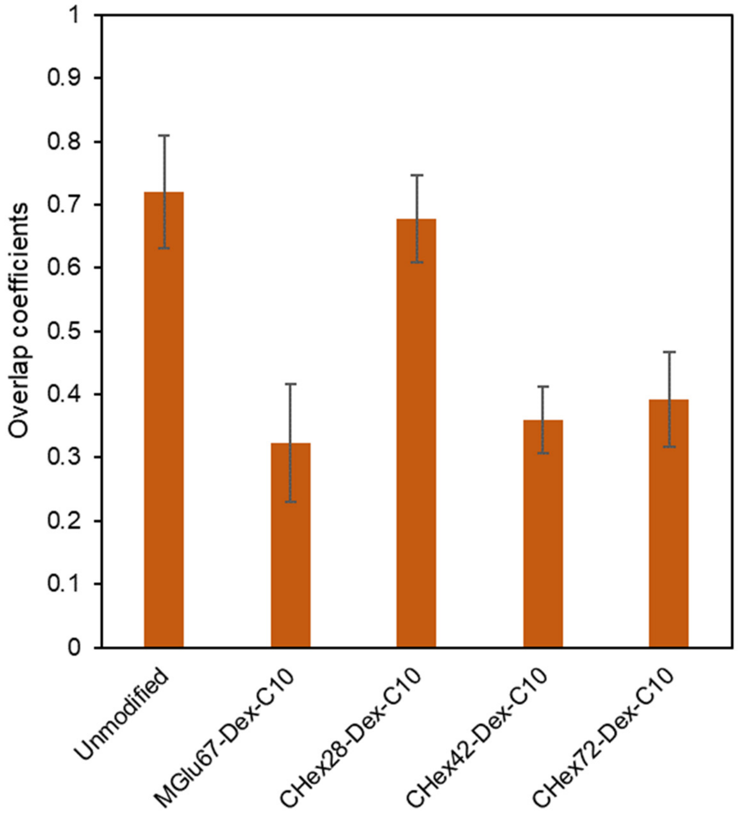

Figure S17. Colocalization for FITC fluorescence derived from FITC-OVA with DiI fluorescence. Overlap Coefficient of FITC fluorescence with DiI fluorescence was calculated from CLSM images.
